# Small Fiber Neuropathy Associated with Hyperlipidemia: Utility of Cutaneous Silent Periods and Autonomic Tests

**DOI:** 10.1155/2014/579242

**Published:** 2014-03-19

**Authors:** G. Morkavuk, A. Leventoglu

**Affiliations:** Ufuk University Medical School, Department of Neurology, Mevlana Bulvarı No. 86-88, Balgat, 06500 Ankara, Turkey

## Abstract

*Background.* Established electrophysiological methods have limited clinical utility in the diagnosis of small fiber neuropathy. The cutaneous silent period (CSP) may be useful as a method for the evaluation of smaller and unmyelinated fiber dysfunctions. Hyperlipidemia is a very rare cause of small fiber neuropathy. In this study, hyperlipidemia and small fiber neuropathy in symptomatic patients with normal nerve conduction studies were evaluated with autonomic tests and cutaneous silent periods. * Methods.* Twenty-five patients with clinically suspected small fiber neuropathy and 23 healthy volunteers were included. CSP latency and duration, as well as CSP latency difference of the upper and lower extremities, were examined. Two tests were used to assess the autonomic nervous system, namely, the *R*-*R* interval variation test in basal and profound breath conditions and the sympathetic skin response. *Results.* Twenty-five patients with clinically suspected small fiber neuropathy and normal nerve conduction studies were compared with 23 controls. In the upper extremities, patients had prolonged CSP latencies (*P* = 0.034) and shortened CSP durations (*P* = 0.039), whereas in the lower extremities, patients had shortened CSP durations (*P* = 0.001). The expiration-to-inspiration ratios were also reduced in patients groups. There was no significant difference between sympathetic skin response latencies and amplitude of the case and control groups. *Conclusion.* Our findings indicate that CSP may become a useful technique for the assessment of small fiber neuropathy in hyperlipidemic patients.

## 1. Introduction

Small fiber neuropathy (SFN) can be defined as generalized peripheral neuropathy, where small myelinated A-delta and unmyelinated C nerve fibers are specifically more affected alone or compared with large fibers. Patients with SFN refer to the neurology clinics with generally positive sensory complaints such as burning, stinging and pain in the feet, and/or autonomic symptoms. The neurological examination in SFN is either completely normal or just impaired pain-temperature sensation is found.

A number of methods are required for early detection and treatment of SFN. The methods for assessing small fiber dysfunction are limited despite its clinical significance. Their clinical use is limited, since most of these methods are invasive or time consuming or require special equipment.

While mainly pain and temperature sensations are affected in small fiber neuropathy, the manifestation may also be accompanied by autonomic dysfunction. In addition, routine nerve conduction studies showing the large fiber functions are within the normal limits.

Cutaneous silent period is an inhibitory spinal reflex characterized by a short-term interruption in voluntary muscle activity following a strong stimulation of a sensory nerve in the skin. There is strong evidence suggesting that the afferent arm and leg of the CSP are formed by somatic small fibers (A-delta) [1–3]. Thus, the CSP might be useful for the functional assessment of somatic small fibers.

The etiology of SFN includes toxic, inflammatory/infectious, hereditary causes, amyloidosis, nutritional, and metabolic such as diabetes mellitus, impaired glucose tolerance, Vitamin B1 and B6 deficiency, and hyperlipidemia. The association of lipid abnormalities and peripheral neuropathy has been reported in many reports [4–8]. Only few reports have suggested the correlation between hyperlipidemia and SFN [7].

The aim of this study is to determine small fiber dysfunctions with CSP, sympathetic skin response, and* R*-*R* interval in hyperlipidemic patients and to compare these results between hyperlipidemic patients and asymptomatic controls.

## 2. Materials and Methods 

### 2.1. Study Population

The study population consisted of hyperlipidemic patients and healthy volunteers. Inclusion and exclusion criteria were applied to patients. The informed consent of patients for electrophysiological testing was obtained from all participants before inclusion.

Forty-eight subjects, consisting of 25 patients (12 females and 13 males) fulfilling the above-mentioned inclusion and exclusion criteria and 23 healthy controls (11 females and 12 males), were included in the study. A detailed medical history was obtained and systemic and neurologic examinations were performed. Patients were excluded if they had a history of any specific peripheral nerve, muscle disease, neuromuscular junction disease, cervical spondylosis, spine surgery, central nervous system disease, including stroke, dementia, or medical conditions associated with peripheral neuropathy, such as DM, metabolic disorders, alcohol abuse, and malignancy.

All of the patients were evaluated in terms of age, sex, weight, body mass index, history of hypertension and diabetes, smoking, fasting plasma glucose and second-hour plasma glucose after a meal, and lipid profile, including, total cholesterol, triglyceride, LDL-cholesterol and HDL-cholesterol, and electrocardiogram. Laboratory investigations included complete blood count, renal and liver function tests, thyroid function tests, vitamin-B12 level, folic acid level, erythrocyte sedimentation rate, and rheumatoid factor. All the patients were fully examined by means of neurological examination and autonomic findings, that is, evaluations for heart rate, blood pressure. An examiner evaluated each patient with hyperlipidemia using the Michigan Neuropathy Screening Instrument (MNSI) [9], Michigan Autonomic Symptom Screening (MASS), Neuropathy Symptom Score (NSS) [10], and DN4 test [11]. The study protocol was in compliance with the Helsinki Declaration of Human Rights and approved by the Ethics Committee of Ankara University, and all the participants provided written informed consent.

### 2.2. Electrophysiological Evaluation

All electrophysiological data were recorded using a Medelec Synergy EMG machine (MEDELEC Synergy, USA) in the electrophysiology laboratory in the Ufuk University Medical Faculty Department of Neurology.

### 2.3. Nerve Conduction Study (NCS)

Each patient's skin temperature was confirmed to be ≥32°C on the dorsum of the hands and feet. Conventional surface electrode techniques were used for each nerve conduction study. All the patients and controls, motor conduction studies were performed from the bilateral common peroneal and posterior tibial nerves, and sensory conduction was studied in the bilateral sural, superficial peroneal nerves in the lower extremities [12]. In the upper extremities, motor and sensory nerve conduction study in median and ulnar nerves were evaluated. Sensory nerves were studied orthodromically in upper extremities. Bilateral sural nerve conductions were evaluated antidromically. Latencies, amplitudes, and velocity parameters were determined for motor and sensory nerves. The latency of the sensory nerve action potential (SNAP) was measured to peak of the negative deflection and used to calculate the conduction velocity. Compound muscle action potentials (CMAP) and SNAP amplitudes were measured from the positive peak to the negative peak using supramaximal percutaneous nerve stimulation with surface recordings. The latencies for compound muscle action potentials were determined as the onset of the negative deflection from the baseline, and the latencies of the sensory action potentials were determined as the negative peak. Filter setting were 20 Hz–10 kHz for motor studies and 20 Hz–2 kHz for sensory studies.

### 2.4. Heart Rate Variability in Response to Deep Breathing (Expiration to Inspiration Ratio (*E/I*))

The expiration to inspiration (*E/I*) ratio is recommended to be sufficient for the evaluation of cardiac autonomic neuropathy [13]. Recordings were made in the morning after subjects were sufficiently relaxed. After giving proper instructions and sufficient training, the subjects were made to lie in supine position and through verbal signal they were asked to breathe maximally allowing five seconds for inspiration and five seconds for expiration for one minute. The parasympathetic test employed in this study was heart rate response to deep breathing at 6 respiratory cycles per minute. The average of five recordings at rest was termed as* R*% and that of two recordings during deep breathing as* D*%. The difference between* D*% and* R*% (*D*-*R*) and the ratio of* D*-*R*% (*D*/*R*) were also calculated.

### 2.5. Sympathetic Skin Response

The test were performed with the subject supine and relaxed in a semidarkened room, in room temperature controlled at 25 to 26°C (skin temperature was maintained at 32°C). The skin temperature was measured and if under 32°C, the limbs were warmed. A standard active electrode was attached to the palm and sole and the reference electrode to the dorsum of the hand and foot. The stimuli used were single electrical stimulus at the wrist contralateral to the recording side [14]. Stimuli were delivered unexpectedly and in irregular intervals of more than 1 min to prevent habituation. The latency was measured from the onset of the stimulus artifact to the onset of the first negative deflection and expressed in seconds. The amplitude was measured from the baseline to the negative peak and expressed in mV. The response was considered absent if no consistent voltage change occurred using a sensitivity of 50 *μ*V per division after three trials at maximum stimuli intensity. In our study, the amplitudes were not included in the analysis because the amplitudes had extent variability even in the same subject in repeated measurements due to possible habituation phenomena. Response latencies were considered pathological when more than 2 SD above the mean latency of the control group.

### 2.6. CSP Evaluation

**Figure 1:**
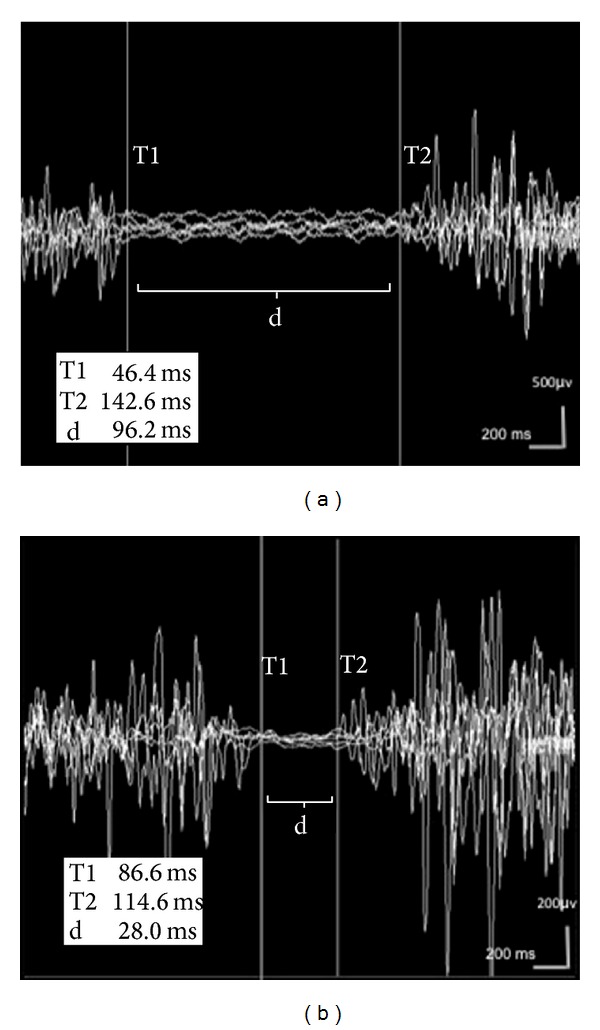
(a) CSP recording from APB muscles in control subject; (b) in hyperlipidemic patient, prolonged CSP latency with reduced duration was showed in the APB muscles recording. (T1, CSP latency; T2, end of the CSP duration, d, CSP duration).

The CSP was recorded in the right upper and the lower extremities. Filters were 50 Hz–5 kHz, sweep speed was 200 ms, and sensitivity was 100 *μ*V. The median sensory nerve was stimulated with a standard painful stimulus (25 mA intensity, 1 ms duration) through a bar electrode fixed on the second digit of the right hand and the response was recorded with an electrode fixed on the belly of the contracting abductor pollicis brevis muscle (Figure 1). The sural nerve was stimulated superficially lateral to the external malleolus in the right lower extremity and recordings were obtained from the anterior tibial muscle through bar electrode [15, 16].

### 2.7. Statistical Analyses

Statistical analyses were performed using SPSS 18.0 for Windows (SPSS Inc., Chicago, IL, USA). Normally distributed data were analyzed by parametric tests (*t*-test and *t*-test for dependent samples). The gender distribution of the two groups was assessed by a chi-square test. CuSP latency and duration were established as the mean from four recordings. The mean, median, standard deviation, and minimal and maximal values were calculated. Student's paired test, Mann-Whitney *U*, chi-square test, and analysis of variance (ANOVA) test were used for comparisons. For correlation analysis, Spearman's rank correlation coefficients were used. Statistical significance level was accepted as *P* < 0.05.

## 3. Results

This study included 25 patients with neuropathic complaints (13 men and 12 women; mean age, 39.1 years) and diagnosed with isolated hyperlipidemia due to the absence of any disease that could cause polyneuropathy and whose routine nerve conduction studies were normal, and 23 healthy subjects with no disease (12 men and 11 women; mean age, 36 years). We recruited 25 patients from the hyperlipidemia clinic having a LDL >130 mg/dL, triglyceride above 150 mg/dL, and total cholesterol >200 mg/dL with SFN symptoms. There were no statistically significant differences in age and gender between the patient and control groups. Body mass index of study group was significantly higher compared to control (*P* = 0.001). Total cholesterol, triglycerides, and LDL-cholesterol were significantly higher in patient group compared to healthy control (*P* = 0.001). In the patient group, significant positive correlation was found between BMI and cholesterol and triglycerides levels. There was no significant difference in HDL-cholesterol and blood pressure between study and control group.* R*-*R* interval, sympathetic skin response (in four extremities), and cutaneous silent period (in abductor pollicis brevis and tibialis anterior muscles) parameters were analyzed in all patient and control group. The clinical characteristics of the subjects are shown in Table 1.

Prolonged CSP latency with reduced duration was showed in the abductor pollicis brevis muscles in the patient (Figure 1). The results indicated that upper extremity cutaneous silent period latency was longer and the duration was shortened in the patient group compared with the control group (*P* = 0.034, *P* = 0.039, resp.) while no statistically significant difference was found for cutaneous silent period latency in the lower extremities between two groups, when the correlation between LDL and total cholesterol level and cutaneous silent period duration in the lower extremity, a negative correlation was found between two groups; in other words, it was found that cutaneous silent period duration in the lower extremity shortened as the LDL and total cholesterol levels increased (Table 2). No correlation was found between triglycerides level and cutaneous silent period latency and duration in the upper or lower extremity. Sympathetic skin response could not be achieved in bilateral lower extremities in 5 patients in the patient group, and no statistically significant difference was observed between sympathetic skin response latency and amplitudes in four extremities between the patient and control group (Table 3). And for the* R*-*R* interval parameters, only* E/I* ratio was found statistically significant between two groups.* E/I* ratio was found decreased in the patient group compared with the control group (*P* = 0.02) (Table 4). There were no significant differences in sympathetic skin response, MNSI, MASS and LDL-cholesterol, triglycerides, HDL-cholesterol, and CSP latency between the patient and the control groups (*P* > 0.005).

## 4. Discussion

SFN is a neuropathy selectively involving small diameter myelinated and unmyelinated nerve fibers. Degeneration of small nerve fibers can foretell the progression to a more diffuse neuropathy [17, 18], making the early diagnosis of SFN important for the accurate treatment of patients. Recent studies have also reported that subclinical involvement of distal large sensory fiber can occur in SFN [19, 20]. The clinical picture of an isolated small fiber neuropathy is characteristic, but the diagnosis is not always easy. Previous studies proposed that the CSP is easily used to assess tool for small-diameter neuropathies [16].

Early detection is important in somatic small fiber polyneuropathy and electrophysiological studies on small-diameter fiber functions. However, there are many reasons why early diagnosis is important, some of which is the definition of the diagnosis which can lead to a focused screening on its etiology. Second reason, early disease modifying or symptomatic treatments can be started. Another reason, early diagnosis and awareness of the SFN can increase patients' compliance, which is particularly important in the treatment of neuropathic pain [21].

SFN is often idiopathic and typically presents with peripheral pain with or without symptoms of autonomic dysfunction. The most common cause is diabetes or glucose intolerance. Other possible causes include hyperlipidemia. Dyslipidemia can also cause peripheral nerve damage. Elevated serum triglyceride levels are associated with an increased risk for sensory neuropathy or small fiber neuropathy. Diagnosis is made on the basis of the clinical features, normal nerve conduction studies, and abnormal specialized tests of small fiber function. These tests include assessment of epidermal nerve fiber density as well as temperature sensation tests, sudomotor and cardiovagal testing, and sympathetic skin response. Although the use of the CSP in the diagnosis of somatic small fiber polyneuropathy should be supported with further studies [1–3], the patients with hyperlipidemia electrophysiological demonstration of the existence of CSP do not have any work. In the present study, nerve conduction studies,* R*-*R* interval, and SSR and CSP evaluations were performed in hyperlipidemic patients with somatic SFN symptoms and findings and healthy controls.

The prolonged CSP latency in patients with hyperlipidemic patients compared to healthy controls was similar to previous studies for diabetic patients [22]. Changes in the lower extremity CSP duration in the patients group have been referred to A-delta nerve fiber involvement.

Several theories have been proposed in the literature to explain the possible relationship between lipid disorders and peripheral neuropathy; one of them suggests that the function and structure of the nerve could be affected by abnormal serum lipids by two mechanisms: first, by the action of lipoproteins as enzyme cofactors and as bound intermediate in the biosynthesis of polysaccharide and proteins. Second, abnormal serum lipids could remote nerve infarction over fat embolism or lipid stimulated platelet aggregation [23]. Wiggin et al. reported that in their subjects with mild to moderate diabetic neuropathy, elevated triglycerides correlated with sural nerve myelinated fiber density loss independent of disease duration, age, diabetes control, or other variables [24].

CSP was studied in patients with various sensory neuropathies. Many investigators were able to demonstrate a reduction in CSP duration in patients with SFN [1, 2]. Leis [25] reported one patient with a pure sensory neuropathy causing absent sensory nerve action potentials and recorded prolonged CSP latency. Syed et al. studied 24 patients with Fabry's disease, a rare disease is X-linked lysosomal storage disorder caused by abnormal developed small and large diameter fibers. In these patients CSP was normal in the upper extremity, but CSP of either reduced or increased duration in the lower extremity. These authors concluded that the CSP must be insensitive to SFN in case of mild and moderate impairments [26]. Corsi et al. [27] studied two patients with hereditary sensory autonomic neuropathy. They found that in these patients CSP of reduced duration could be gained when stimuli were applied to two digits. Yaman et al. [28] have reported prolonged CSP latency in 35 patients with diabetic neuropathy compared to controls and they found that CSP duration was shortened and prolonged CSP latency in diabetic patients with small fiber neuropathy. These authors concluded that the CSP may be a useful electrophysiological method for the detection and diagnosis of small fiber neuropathy in diabetic patients. Onal et al. [29] also found similar findings. They found normal CSP in the upper extremity, but CSP of reduced duration and longer latency in the lower extremity. They suggested that the difference was more significant in patients with neuropathic pain. These authors concluded that the CSP evaluation might be used to support the diagnosis in diabetic patients with suspected somatic SFN.

Changes in the upper and lower extremity CSP latency and duration in SFN have been attributed to A-delta nerve fiber involvement. The findings support the association between CSP changes and A-delta nerve fibers. We have also found that the CSP latency is prolonged and the CSP duration is shortened in the lower extremities of hyperlipidemic patients.

## 5. Conclusions

CSP may be a useful electrophysiological method for the diagnosis of small fiber neuropathy in hyperlipidemic patients. Therefore, we believe that it would offer an insight into other studies in the future on diagnosis of SFN due to hyperlipidemia and contribute to the literature.

## Figures and Tables

**Table 1:** Clinical characteristics of patients and control groups.

	Patients (*n* = 25)	Controls (*n* = 23)	*P* value
Age	39.1 ± 9.5	36 ± 9	NS
Gender (male %)	52%	52%	NS
BMI	27.9 ± 5.2	23.1 ± 3.2	0.001*
Total cholesterol (mg/dL)	231.3 ± 44.7	142.6 ± 27.5	0.001*
Triglycerides (mg/dL)	192.5 ± 101.1	86.8 ± 33.9	0.001*
HDL (mg/dL)	46.3 ± 16.3	51 ± 13.6	NS
LDL (mg/dL)	152.5 ± 39.9	82 ± 21	0.001*

Neurological examination findings			
Normal	56%		
Reduced ankle tendon reflex	8%		
Reduced distally vibration sensation	36%		
Reduced touch sensation at the foot	8%		
Sensorimotor symptom,			
Numbness	48%		
Paresthesia/dysesthesia	48%		
Burning pain	44%		
Muscle cramps	76%		
Autonomic symptoms,			
Lightheadedness	40%		
Dry mouth/dry eyes	24%		
Pale/blue feet	4%		
Cold feet	40%		
Decreased/absent sweating/feet	8%		
Nausea, vomiting, after eating a meal	32%		
Persistent diarrhea	4%		
Persistent constipation	4%		
Urinary incontinence	8%		
Erectile dysfunction (male)	0		

NS: no significance; BMI: body mass index; HDL: high density cholesterol; LDL: low density cholesterol, *P < 0.05.

**Table 2:** CSP latency and duration measured from upper and lower extremities of patients and controls groups.

CSP (ms)	Patient group (*n* = 25) mean ± SD	Control group (*n* = 23) mean ± SD	*P* value
Upper extremity			
Latency	69.1 ± 15.4	58.6 ± 16.2	0.034*
Duration	56.6 ± 20.0	67.9 ± 19.9	0.039*
Lower extremity			
Latency	89.9 ± 32.6	88.3 ± 12.3	0.103
Duration	35.7 ± 20.6	55.6 ± 15.6	0.001*

SD: standard deviation; CSP: cutaneous silent period, *P < 0.05.

**Table 3:** Sympathetic skin response, mean latency values in hyperlipidemic patients and controls.

	Patient group (mean ± SD) (*n* = 25)	Control group (mean ± SD) (*n* = 23)	*P* value
Upper limb	1.43 ± 0.1	1.36 ± 0.1	0.18
Sole latency (sec)	1.58 ± 0.8	1.94 ± 0.1	0.56

*P < 0.05.

**Table 4:** Mean *R*-*R* interval variation values in patients and controls.

	Patient group (mean ± SD) (*n* = 25)	Control group (mean ± SD) (*n* = 23)	*P* value
*R*%	25.2 ± 11.6	22.3 ± 8.3	0.489
*D*%	33.2 ± 11.4	33.2 ± 9.6	0.672
*D*-*R*	1.43 ± 0.55	1.56 ± 0.40	0.375
*D*/*R*	1.28 ± 0.16	1.38 ± 0.16	**0.021**

*R*%: *R*-*R* interval variation at rest; *D*%: during deep breathing, *D* − *R*: the difference between *D*% and *R*%; *D*/*R*: the ratio of *D*-*R* %, *P < 0.05.
